# Molecular Investigation of Product Nkabinde in HIV Therapy: A Network Pharmacology and Molecular Docking Approach

**DOI:** 10.3390/ijms27020808

**Published:** 2026-01-13

**Authors:** Samuel Chima Ugbaja, Mlungisi Ngcobo, Siphathimandla Authority Nkabinde, Magugu Nkabinde, Nceba Gqaleni

**Affiliations:** 1 Traditional Medicine, School of Medicine, University of KwaZulu Natal, Durban 4000, South Africa; ngcobom3@ukzn.ac.za (M.N.); nceba5850@gmail.com (S.A.N.); magugun@webmail.com (M.N.); 2 African Health Research Institute (AHRI), 719 Umbilo Road, Durban 4001, South Africa

**Keywords:** traditional medicine, polyherbal formulation, Product Nkabinde, HIV, network pharmacology, molecular docking

## Abstract

HIV/AIDS continues to pose a significant global public health concern, with Sub-Saharan Africa having the highest number of people living with HIV (PLHIV). Traditional medicines have been increasingly essential in treating and managing PLHIV. Product Nkabinde (PN), a polyherbal formulation derived from traditional medicinal plants, has recently demonstrated significant potential in the treatment of HIV. This study aims to elucidate the molecular mechanisms underlying the therapeutic effects of phytochemicals identified from PN in HIV treatment, utilizing network pharmacology and molecular docking. The intersecting (common) genes of the 27 phytochemicals of PN and HIV were computed on a Venn diagram, while the protein–protein interaction (PPI) network of the intersecting genes was plotted using STRING. The hub (10) genes were computed and analyzed for Gene Ontology (GO) and Kyoto Encyclopedia of Genes and Genomes (KEGG) enrichment pathways using ShinyGO. Molecular docking and protein–ligand interaction analysis of the 27 phytochemicals with each of the 10 hub genes were performed using the Maestro Schrodinger suite. The KEGG analysis reveals an important network with lower False Discovery Rate (FDR) values and higher fold enrichment. The pathway enrichments reveal that the 10 hub genes regulated by PN focus on immune regulation, metabolic modulation, viral comorbidity, carcinogenesis, and inflammation. GO analysis further reveals that PN plays key roles in transcription regulation, such as miRNA, responses to hormones and endogenous stimuli, oxidative stress regulation, and apoptotic signalling, kinase binding, protein kinase binding, transcription factor binding, and ubiquitin ligase binding enriched pathways. Consequently, molecular docking unveils complexes with higher binding energies, such as rutin-HSP90AA1 (−10.578), catechin-JUN (−9.512), quercetin-3-O-arabinoside-AKT1 (−9.874), rutin-EGFR (−8.127), aloin-ESR1 (−8.585), and quercetin-3-0-β-D-(6′-galloyl)-glucopyranoside-BCL2 (−7.021 kcal/mol). Overall, the results reveal pathways associated with HIV pathology and possible anti-HIV mechanisms of PN. Therefore, further in silico, in vitro, and in vivo validations are required to substantiate these findings.

## 1. Introduction

HIV and AIDS continue to pose a challenge in global public health despite the recent improvement depicted in Table 1. Sub-Saharan Africa still records the highest number of people living with HIV (PLHIV), as illustrated in Table 1 [1]. In a recent study by our research group on the evaluation of the public health impact of the suspension of foreign aid by the United States government, there is a projected increase in the number of new HIV cases (9,050,000) and HIV-related deaths (6,300,000) by 2029 [2].

The essential use of traditional medicine (TM) in treating various diseases has recently gained global public interest in drug and chemotherapeutic applications. TM has been practical and valuable in rural suburbs where conventional treatment methods are inadequate and readily available [3,4]. There has been a reported increase in the use of TM, which has become increasingly essential in treating and managing PLHIV [5,6].

Product Nkabinde (PN) is a traditional polyherbal formulation of four medicinal plants with patent reference number 2023/03587. The traditional healer (PN intellectual property owner) has been using PN in the treatment and management of HIV and other STIs [7,8,9]. PN polyherbal plants are located in different places around the world, including South Africa (KwaZulu-Natal, Limpopo, and Mpumalanga), Zimbabwe, Mozambique, Ethiopia, the Sudano-Sahelian region of West Africa, and Madagascar [10,11,12]. The leaves, stem bark, roots, and fruits of these polyherbal plants contain polyphenols, tannins, coumarins, flavonoids, triterpenoids, and phytosterols, and are used for medicinal purposes, especially due to their immunomodulatory potential [13,14]. One of the plants has been used as a remedy for bowel infections and to manage HIV and AIDS in Nigeria. A study reported that one of the active compounds (tannins) found in the Anacardiaceae family can block the entry of HIV into cells by interfering with the gp41 six-helix bundle formation [15,16]. Its flavonoids are also exceptional metal chelators because they can block HIV-1 activity in in vitro models [17].

A previous study by our research group, entitled “An in vitro study to elucidate the effects of Product Nkabinde on immune response in peripheral blood mononuclear cells of healthy donors,” reported that PN exhibits in vitro immunomodulatory potentials that could impact immune and inflammatory responses [18]. Another study, entitled “Herbal formulations, Product Nkabinde and *Gnidia sericocephala*, exhibit potent in vitro activity against HIV-1 infection,” further elucidated PN’s anti-HIV potential [19]. The four constituent plants of PN, including *Sclerocarya birrea* stem/leaf, *Gnidia sericocephala* roots, *Senna italica* roots, and *Pentanisia prunelloides* roots, were obtained near Tugela Ferry in Msinga, KwaZulu-Natal by the healers [18,19]. Unveiling the molecular mechanisms underlying the therapeutic effect of PN in HIV treatment requires identifying the different metabolites, active compounds, or phytochemicals that constitute this polyherbal formulation. Previous chemical profiling studies by our collaborators at the University of Pretoria had identified twenty-seven (27) prominent phytochemicals that constitute PN as reported in the literature [20,21]. The present study aims to unveil the molecular mechanisms underlying the therapeutic effects of phytochemicals from PN in HIV treatment using network pharmacology and molecular docking.

## 2. Results and Discussion

### 2.1. Comparative Analysis of Intersecting Gene Sets

**Figure 1:**
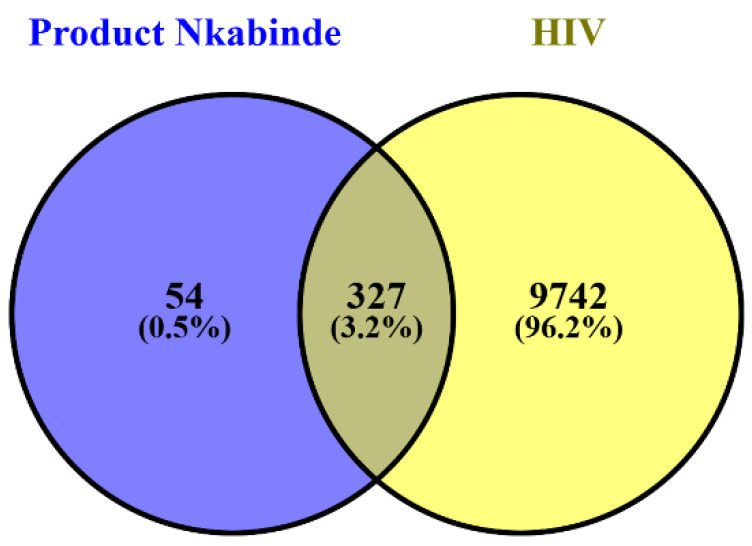
Venn diagram illustrating the overlap between predicted molecular targets of PN phytochemicals and HIV-associated genes retrieved from public databases. The intersecting region represents shared targets used for subsequent protein–protein interaction network construction, enrichment analyses, and molecular docking.

This study aims to elucidate the molecular mechanisms underlying the therapeutic effects of phytochemicals identified from PN in HIV treatment, utilizing network pharmacology and molecular docking. Twenty-seven prominent phytochemicals from PN were used to conduct a comparative analysis of PN and HIV gene sets. The resultant Venn diagram was drawn, and 327 common genes for HIV and PN phytochemicals were identified, as shown in Figure 1. This figure depicts 54 (0.5%) unique gene targets for PN that are not associated with HIV, marked in the blue region. There are 9742 (96.2%) specific HIV genes not linked to PN phytochemicals, shown in the yellow region. The 327 (3.2%) common or intersecting genes linked to PN and HIV indicate shared treatment targets. These 327 intersecting gene targets constitute the primary network for further investigation, including PPI, KEGG, GO, molecular docking, and protein–ligand interaction analysis.

### 2.2. Protein to Protein Interactions

**Figure 2:**
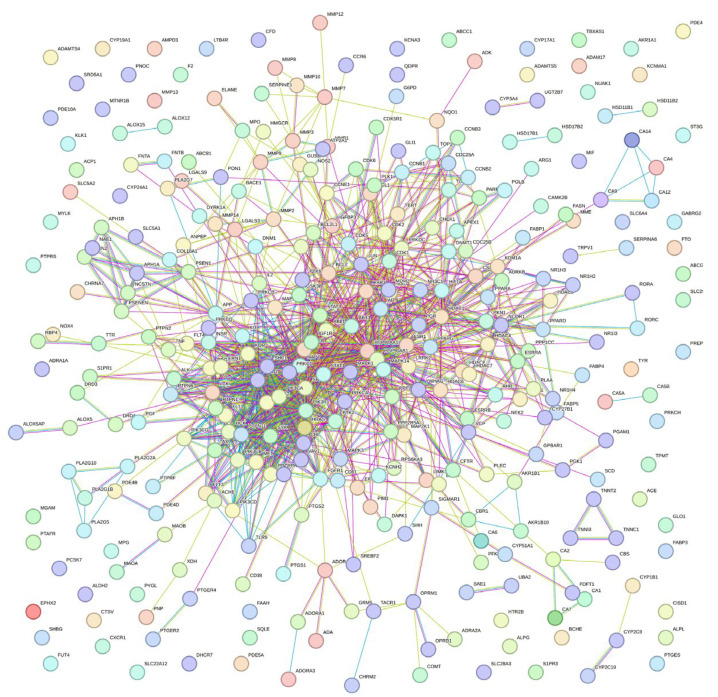
Protein–protein interaction (PPI) network of intersecting targets between Product Nkabinde (PN) phytochemicals and HIV-associated genes. Nodes represent proteins, while edges indicate experimentally supported or predicted interactions.

The PPI network, as shown in Figure 2, indicates PN-HIV interactions with a high degree of confidence, facilitating further investigation into PN-HIV-associated biological processes. The high interconnection of the multiple networks reveals a highly dense key protein at the centre, with outwardly spread nodes, suggesting that PN exerts multi-targeted pathways and effects in HIV therapy. Figure 2 further illustrates that PN has multifaceted and multitargeted HIV-related proteins, whose primary biological activities are related to immune function and infection. The identification of hub genes and further investigation promises to unveil the enrichment analysis of GO and KEGG pathways.

### 2.3. Identification of the Hub Genes

**Figure 3:**
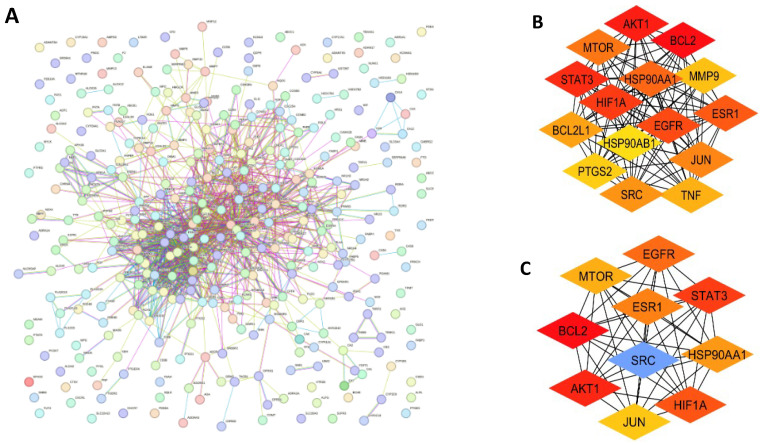
Representation of the 327 genes (**A**), 15 hub genes (**B**), and 10 hub genes (**C**) for the PN-HIV PPI Network.

For the identification of the hub genes from the 327 PN-HIV genes, an initial prediction of fifteen (15) and ten (10) hub genes was carried out at the CytoHubba of the Cytoscape application software program using the MCC and the degree topological analytic method. The MCC is used to measure the maximum cliques; a node of a gene or protein is essential in network stability and function, indicating a pivotal biological process. It is highly sensitive and accurate in identifying the hub genes in dense and highly interconnected networks. Consequently, degree centrality measures the direct number of interconnections among different nodes. The higher the nodes, the better the direct connections. However, degrees are used in preliminary selection, but they are less sensitive and less accurate than MCC because they sometimes omit essential context specificity [22,23]. Figure 3 depicts the preliminary 15 hub genes (Figure 3B) and the final 10 hub genes (Figure 3C) predicted from the 327 PN-HIV intersecting genes (Figure 3A). The selected 10 hub genes play important roles in the HIV infection cycle and were further subjected to GO and KEGG enrichment pathway analysis.

The identified PN-HIV 10 intercepting hub genes include Proto-Oncogene Tyrosine-Protein Kinase Src (SRC), Epidermal Growth Factor Receptor (EGFR), Mechanistic Target of Rapamycin (MTOR), Signal Transducer and Activator of Transcription 3 (STAT3), Estrogen Receptor 1 (ESR1), Heat Shock Protein 90 Alpha Family Class A Member 1 (HSP90AA1), Hypoxia-Inducible Factor 1-Alpha (HIF1A), Jun Proto-Oncogene, AP-1 Transcription Factor Subunit (JUN), AKT Serine/Threonine Kinase 1 (AKT1), and B-Cell Lymphoma 2 (BCL2) as depicted in Figure 3C. These hub genes, their functions, and implications in HIV pathogenesis are depicted in Table 2 below.

### 2.4. Functional Enrichment Analysis

**Figure 4:**
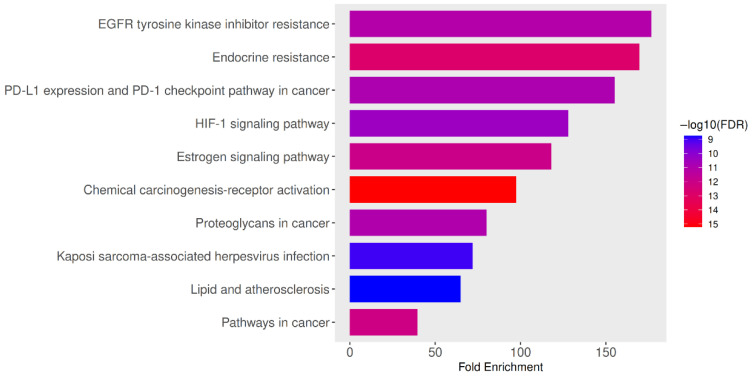
KEGG pathway enrichment analysis of intersecting PN-HIV targets is shown as a bar chart. Enriched pathways include PI3K–Akt, MAPK, apoptosis, HIF-1, TNF, FoxO, NF-κB, p53, estrogen signaling, EGFR tyrosine kinase inhibitor resistance, pathways in cancer, viral carcinogenesis, and HIV-1 infection.

**Figure 5:**
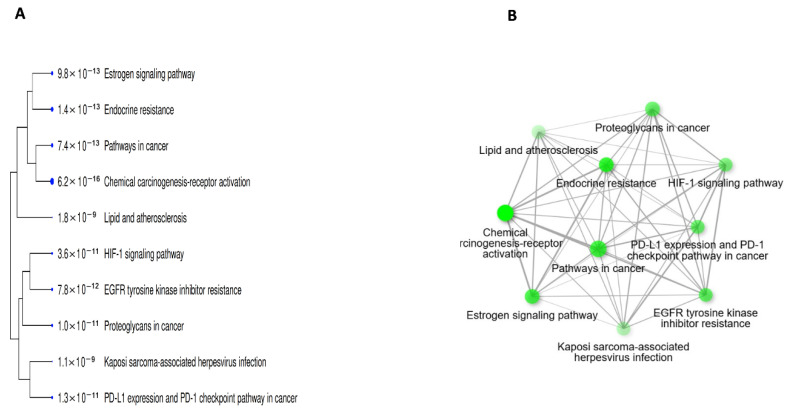
Diagram of the Tree (**A**) and Network (**B**) of the pathways fold enrichment.

**Figure 6:**
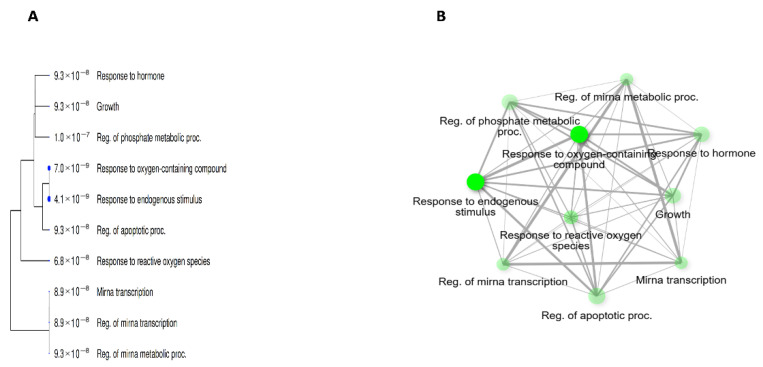
Gene Ontology Biological Process Pathways showing the Tree (**A**) and Network (**B**).

**Figure 7:**
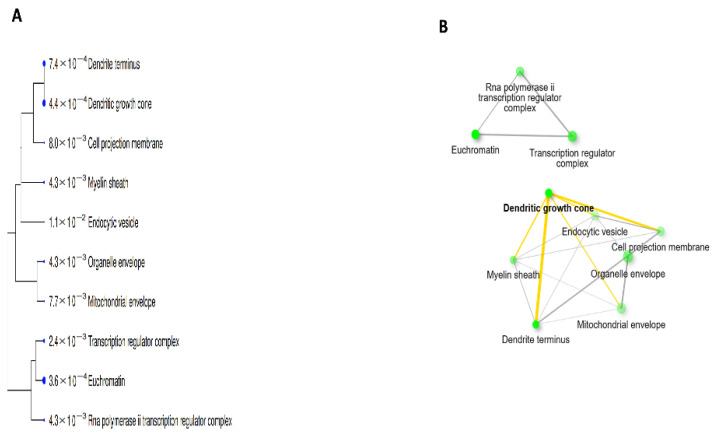
Gene Ontology Cellular Component Pathways showing the Tree (**A**) and Network (**B**).

**Figure 8:**
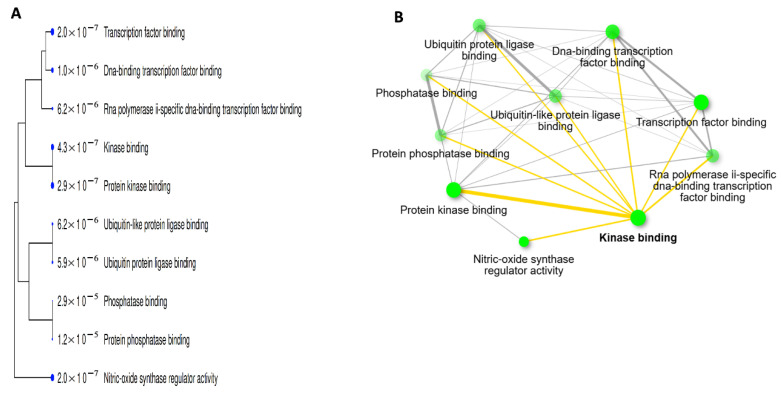
Gene Ontology Molecular Function Pathways showing the Tree (**A**) and Network (**B**).

Functional enrichment analysis was conducted using the FDR cut-off (0.05), with a pathway size minimum of 2 and a maximum of 2000, to show the 10 most important PN-HIV. In this study, under the pathway database menu, all available gene sets, GO biological process, GO cellular component, GO molecular function, and KEGG were chosen and analysed separately. The findings are discussed and presented in Table 3 and Figure 4, Figure 5, Figure 6, Figure 7 and Figure 8 below.

Table 3 presents the KEGG pathway enrichment analysis of the PN-HIV 10 hub genes, indicating a robust mechanistic association that underscores the therapeutic significance of these genes in HIV treatment with PN. The critical network, having lower FDR values and higher fold enrichment, suggests that the 10 hub genes are strongly implicated in cellular processes that intersect with HIV pathogenesis and immunomodulation. For example, the respective 177.6- and 169.6-fold enrichment for EGFR tyrosine kinase inhibitor resistance and endocrine resistance suggest processes in cell viability, replication, and resistance signalling pathways employed by HIV, which PN could assist in normalizing and improving the host cellular resilience. This is supported by a study that revealed the HIV-1 matrix protein p17 stimulates angiogenesis in brain endothelial cells through EGFR-mediated signaling [47]. Additionally, EGFR mutations were examined in HIV-infected individuals with lung adenocarcinoma, comparing HIV-positive and HIV-negative groups [48]. The 155.1-fold enrichment of the PD-L1/PD-1 checkpoint pathway plays a crucial role in immune suppression in PLHIV, and its enrichment suggests that PN has the potential to restore T-cell function, thereby reducing viral immunosuppression. Examination of immune checkpoint blockade in HIV elucidates the role of PD-L1/PD-1 pathways in HIV persistence and immunological depletion [49]. Consequently, a study indicating soluble checkpoint proteins (including PD L1) are related to non-AIDS occurrences in PLHIV has been reported [50].

A study by Duette et al. [51], entitled “Induction of HIF 1α by HIV 1 infection in CD4+ T cells,” shows that HIV-1 upregulates HIF-1α, with consequences for viral replication and extracellular vesicles [51]. The 128-fold and 118-fold increases in the HIF-1 signalling pathway and estrogen signalling are associated with metabolic adjustment, low oxygen adaptation, and hormonal balance, which is common to immunomodulation and HIV coinfection. In addition, another review on HIV 1 infection and glucose metabolism reprogramming via HIF-1α further supports the above result [52]. A high enrichment of 97.4-fold (chemical carcinogenesis-receptor activation), 80.2-fold (proteoglycans in cancer), and 39.6-fold (pathways in cancer) depicts the intersection among HIV-associated inflammation, carcinogenesis, and the host gene expression interruption. Previous studies on inflammation and cardiovascular disease, insulin resistance, and lipid abnormalities link HIV, lipid metabolism, and comorbidity pathways [53,54]. This further suggests that PN could intervene in HIV-associated cancer when these common pathways are targeted. More importantly, the 71.9-fold enrichment of Kaposi sarcoma-associated herpesvirus infection reveals how the 10 hub genes overlap with immunocompromised-related infections common in PLHIV, thereby suggesting that, in addition to viral load reduction, PN may also have preventive effects. Furthermore, the 64.9-fold (lipid and atherosclerosis pathways) suggests therapeutic potential in preventing HIV-related heart disease, a major coinfection in long-term PLHIV. Altogether, the above pathway enrichments reveal that the 10 hub genes regulated by PN focus on immune regulatory pathways, metabolic modulation, viral comorbidity, carcinogenesis, and inflammation, all of which are associated with HIV pathology. Moreover, previous studies have reported how HIV is a major cofactor in Kaposi’s sarcoma-associated herpesvirus infection and the development of Kaposi’s sarcoma, how HIV 1 Tat protein interacts with Kaposi’s sarcoma-associated herpesvirus latency antigen in co-infected cells, and that high Kaposi’s sarcoma-associated herpesvirus seroprevalence in HIV-infected adults and associations with advanced HIV disease [55,56,57]. Therefore, PN is not proposed as a substitute for ART, but rather as a preclinical candidate that requires extensive validation before any clinical consideration. The nGenes associated with each pathway are indicated in red as computed with the ShinyGO 0.85 database.

Furthermore, the bar plot in Figure 4 and the illustrations in Figure 5A,B show that the 10 hub genes from the PN–HIV network are highly enriched in pathways related to drug resistance, including EGFR tyrosine kinase inhibitor resistance, endocrine resistance, and the PD-L1 expression and PD-1 checkpoint pathway in cancer. The higher fold enrichment and –log10 (FDR) values indicate strong enrichment with very high statistical significance, as lower FDR values are directly proportional to higher confidence. This projection further suggests that PN could impact HIV therapy through host-targeting approaches and signalling pathways implicated in resistance, immunomodulatory, and viral coinfection.

The Gene Ontology Biological Process Pathways, as shown in Table 4 and Figure 6A,B below, demonstrate that PN phytochemicals affect major cellular functions crucial in HIV therapy. The enriched pathways include regulation of transcription, such as miRNA, responses to hormones and endogenous stimuli, regulation of oxidative stress, and apoptotic signaling. These pathways further reveal that PN can enhance immunomodulation, reduce HIV-related oxidative stress, and promote regulated cell death, thereby complementing antiviral therapy. These findings have been previously reported in the literature. A report by Morando et al. [58] entitled ‘The Role of MicroRNAs in HIV Infection’ places miRNAs as important factors that affect how the host and virus interact, which opens up new ways to improve the diagnosis, treatment, and prevention of HIV infection and supports the transcription/miRNA and transcription factor regulation claims [58]. Ivanov et al. [59] reported on ‘Oxidative Stress during HIV Infection: Mechanisms and Consequences’, which supports the findings on oxidative stress regulation, apoptosis links, and inflammation [59].

The Gene Ontology cellular component pathway, as shown in Table 5 and Figure 7A,B below, demonstrates that the PN disrupts HIV-associated cellular components and host immune response mechanisms. The major cellular components include dendritic growth cones, endocytic vesicles, mitochondrial and organelle envelopes, and transcription regulator complexes. The pathway network further underscores that PN could modulate viral endocytosis, modulate immune signalling pathways, regulate host transcription and energy metabolism, which interferes with HIV replication and enhances cellular resistance. These findings are supported by previous research and reported in the literature [60,61,62,63,64,65,66].

The Gene Ontology molecular function pathways, as shown in Table 6 and Figure 8A,B, indicate that PN is primarily involved in regulating and essential binding processes during HIV pathogenesis. The enriched functions include kinase binding, protein kinase binding, transcription factor binding, and ubiquitin ligase binding, all of which are crucial in regulating viral replication, immune signaling, and viral infection mechanisms. PN modulates kinase activity and transcription factor interactions, thereby interfering with HIV’s dependence on host cell signalling. Moreover, PN could regulate ubiquitination and nitric oxide synthase mechanisms, suggesting immunomodulatory and antiviral functions. This further highlights PN’s multiple targets and impacts, which interfere with the main processes involved in HIV resistance and have been previously reported in the literature [67,68,69].

### 2.5. Molecular Docking and Protein–Ligand Interaction Analysis

The molecular docking and ligand–protein interaction of each of the 27 phytochemicals of PN with the PN-HIV 10 hub genes were computed and analysed. The binding energies (kcal/mol) of the top five (5) complexes are shown in Table 7 below.

Table 7 above shows a broad range of interactions between PN phytochemicals and the 10 PN-HIV hub protein targets. The complexes of SRC, MTOR, HSP90AA1, and BCL2 with catechin, rutin, gallic acid, procyanidin B2, quercetin derivatives, and 7-7′-dihydroxy-3-8′-biscoumarin, in some cases, show a higher or comparable binding energy by PN phytochemicals relative to co-crystallized ligands, suggesting effective inhibition or modulation of HIV pathways. Conversely, some of the ligand–protein complexes displayed lower binding energies comparable to the reference co-crystallized ligands in some proteins, including −8.585 vs. −11.658 (ESR1), −9.874 vs. −11.385 (AKT1), −9.512 vs. −10.563 (JUN), and −6.253 vs. −12.045 (STAT3) kcal/mol, respectively, suggesting relatively lower binding energies. However, the relatively lower binding energies displayed by these complexes do not indicate poor inhibition in the context of PN, a polyherbal formulation. For example, the differences of −7.153 vs. −8.011 kcal/mol in catechin-HIF1A and −8.450 vs. −11.658 kcal/mol in rutin-ESR1 complexes suggest polyherbal synergistic contributions of PN, indicating its therapeutic potential in HIV treatment. As a polyherbal formulation, PN exhibits a multitarget approach, which is essential in HIV treatment, where disruption of viral replication and pathogen–host interactions is required. Consequently, the complexes with higher binding energies, such as rutin-HSP90AA1, catechin-JUN, quercetin-3-O-arabinoside-AKT1, rutin-EGFR, aloin-ESR1, and quercetin-3-0-β-D-(6′-galloyl)-glucopyranoside-BCL2, should be given priority in further in vitro and in vivo validations.

Protein–ligand interaction analysis further reveals the protein active site residues involved in the binding, the hydrogen bond network, and the ionic and hydrophobic interactions responsible for the complex stability (Table 8; also presented in Supplementary Figures S1–S6).

## 3. Materials and Methods

### 3.1. Compound Retrieval and Screening

In this study, the SMILES of the 27 prominent phytochemicals identified through chemical profiling from PN were retrieved from PubChem in August 2025. The gene targets for each phytochemical were then predicted using the Swiss Target Prediction and Similarity Ensemble Approach (SEA) database [70,71,72]. A total of 1034 genes were retrieved and saved in an Excel spreadsheet. The gene dataset was cleaned, and 390 genes were used for further investigation. The human genes associated with HIV were explored and retrieved from GeneCards, a comprehensive human gene database that contains detailed information on all annotated gene sets related to diseases [73]. A total of 10,074 genes were retrieved and saved in an Excel spreadsheet. After data cleaning, 10,068 gene datasets were used for further analysis.

### 3.2. Comparative Analysis of Product Nkabinde and HIV Gene Sets

The intersecting genes between PN and HIV were computed using VENNY 2.1.0, which involves drawing the Venn diagram of the common gene targets. The datasets for HIV and PN phytochemicals are uploaded differently in VENNY 2.1.0 and are named accordingly on the bioinformatics & evolutionary genomics webpage [74]. The Venn diagram is drawn, and 327 common genes for both are identified for further analysis.

### 3.3. Protein–Protein Interaction (PPI) Network Analysis

The 327 common Gene Sets were used to construct the protein–protein interaction (PPI) network, enabling us to get the interactions with a high degree of confidence for further studies. Search Tool for the Retrieval of Interacting Genes (STRING) database was used for the PPI analysis [75]. In the STRING database, the multiple proteins icon was selected, followed by uploading the 327 common genes after selecting Homo sapiens from the drop-down menu, and the PPI network was computed. The analysed PPI network was exported and saved in high-resolution Portable Network Graphics (PNG).

### 3.4. Identification of the Hub Genes

The PPI network was further exported to Cytoscape to identify hub genes [76]. The hub genes are sometimes referred to as key genes that are involved in relevant biological processes, ensuring the stability of the PPI network. They regulate cell functions, unveiling the mechanistic dynamics of diseases. The latest version of Cytoscape, 3.10.3, was downloaded. Cytohubba and yfile were then installed in the app. Cytohubba in Cytoscape was opened while the 327 PPI network was exported from STRING to determine hub genes. A preliminary selection of ten (10) hub genes was done using the Maximal Clique Centrality (MCC) and degree topological analysis techniques to rank the hub genes subject to centrality and importance in the PPI network [22,23].

### 3.5. Functional Enrichment Analysis

The ShinyGO 0.85 database was utilized for Kyoto Encyclopaedia of Genes and Genomics (KEGG) and the Gene Ontology (GO) pathway analysis [77]. The 10 hub genes were uploaded into the database and analysed to identify their functional enrichment, enabling understanding of their biological functions, potential treatment targets, and unveiling the mechanistic processes of the disease (HIV) [78,79]. The computation of *p*-values and False Discovery Rates (FDR) is done using the hypergeometric test and the Benjamini–Hochberg technique [80]. The fold enrichment is computed as the percentage of genes in the 10 hub genes that are in a pathway divided by the corresponding percentage in the background genes. The fold enrichment is a direct measurement of the effect size, while FDR indicates the statistical significance [81]. In this study, an FDR cut-off of 0.05 was used to show the 10 most useful pathways. The average sorting of the pathways is done using FDR and fold enrichment when ‘Select by FDR and Sort by Enrichment’ was selected.

### 3.6. Protein and Ligand Preparations

The X-ray crystalized structures of the 10 hub genes/proteins were retrieved from the Research Collaboratory for Structural Bioinformatics Protein Data Bank (RCSB PDB) with PDB IDs 8B83 (SRC), 4Y46 (JUN), 1PBK (MTOR), 4R3M (HSP90AA1), 1XP1 (ESR1), 4R3P (EGFR), 2ILM (HIF1A), 6NUQ (STAT3), 3O96 (AKT1), and 4AQ3 (BCL2) [82]. Furthermore, protein IDs were retrieved, imported, and pre-processed using the Protein Preparation Wizard suite in Maestro. The binding sites were generated using the receptor grid generation [83]. Ligprep was used to import the 27 phytochemicals into the project table. The OPLS4 force field was used in preparing the ligands, along with each of the co-crystalized ligands in the retrieved PDB IDs, prior to molecular docking.

### 3.7. Molecular Docking and Protein–Ligand Interaction Analysis

Molecular docking and protein–ligand interaction analysis were computed using the Schrodinger Maestro suite to predict and evaluate binding energies for the predicted PN-HIV hub genes [83]. Molecular docking reveals the likely multi-targeted molecular processes of the PN-phytochemicals that consistently align with the network pharmacology structure. Molecular docking further reveals multiple therapeutic targeted networks rather than single-targeted ones [84,85]. Protein–ligand interactions reveal the protein active site residues involved in binding, the hydrogen bond network, and the ionic and hydrophobic interactions responsible for a stable and specific PN-HIV protein network, highlighting the synergistic roles in inhibiting HIV replication and disease progression. The Schrödinger Maestro Suite was used for molecular docking. The RCSB Protein Data Bank provided high-resolution human X-ray crystal structures of the ten PN–HIV hub proteins (SRC, EGFR, MTOR, STAT3, ESR1, HSP90AA1, HIF1A, JUN, AKT1, and BCL2), co-crystallized with reference ligands. These structures were then prepared using the Protein Preparation Wizard by assigning bond orders, adding hydrogens, optimizing protonation states at physiological pH (7.0 ± 0.2) using Epik, refining hydrogen-bond networks, and using the OPLS4 force field to minimize energy beyond 5 Å from the binding site. To guarantee biologically appropriate docking, receptor grids were created by focusing on the co-crystallized ligand binding sites. After obtaining the 27 PN’s phytochemicals from PubChem, LigPrep was used to generate the proper ionization states, tautomers, and stereoisomers at a pH of 7.0 ± 2.0. OPLS4 geometry optimization was then performed. Glide was used for docking in a hierarchical approach, where ligands were treated as flexible, and receptors were kept stiff. Standard Precision (SP) docking was used for preliminary screening, while Extra Precision (XP) docking was used for improved pose selection. GlideScore (kcal/mol), which integrates hydrogen bonding, hydrophobic, electrostatic, and desolvation effects, was used to rank binding affinities. By re-docking the co-crystallized ligands and verifying the conservation of binding orientation and important interaction residues, the docking methodology was validated. To identify stabilizing hydrogen bonds, hydrophobic contacts, π–π interactions, and charged interactions within the active sites, protein–ligand interactions were then examined using Maestro [86,87].

## 4. Conclusions

Traditional medicine has been practical and valuable in rural areas where conventional treatment methods are inadequate and not readily available. There has been a reported increase in the use of TM, which has become increasingly essential in treating and managing PLHIV. PN, a traditional polyherbal formulation of four medicinal plants, has been used in the treatment and management of HIV and other STIs in South Africa. The study of the 10 hub genes, out of the 327 common genes shared between PN and HIV, unveils immune regulatory pathways, metabolic modulation, viral comorbidity, carcinogenesis, and inflammatory potential of PN, all of which are associated with HIV pathogenesis. Molecular docking reveals the likely multi-targeted molecular processes of the PN-phytochemicals that consistently align with the network pharmacology structure. Molecular docking further reveals multiple therapeutic targeted networks rather than single-targeted ones. However, polyphenols, including those identified in PN, are known to exhibit pleiotropic bioactivity due to their ability to interact with multiple protein targets, which may result in both therapeutic and off-target effects. While this multi-target nature underpins their potential efficacy in complex diseases such as HIV, it may also lead to unintended modulation of host signalling pathways at higher concentrations or prolonged exposure. Recent studies have highlighted that the off-target effects of polyphenols are often context- and dose-dependent, and are mitigated by their generally favorable safety profiles and metabolic clearance in vivo [88,89]. Consequently, the present findings should be interpreted as preclinical evidence, underscoring the need for further pharmacokinetic, toxicity, and selectivity studies to distinguish between beneficial and off-target interactions before any clinical consideration. Therefore, further in silico, in vitro, in vivo, and human trials are recommended to validate the findings of this present study.

## Figures and Tables

**Table 1:** Global and Regional HIV Statistics (2024/Reported 2025) [1].

Region	PLHIV (Millions)	New HIV Infections (Thousands)	AIDS-Related Deaths (Thousands)	Antiretroviral Therapy (ART) Coverage (%)
Eastern & Southern Africa	21.1	490	260	84
Western & Central Africa	5.2	160	120	76
Asia & Pacific	6.9	300	150	69
Latin America	2.5	120	27	71
Caribbean	0.34	15	4.8	74
Eastern Europe & Central Asia	2.1	130	48	51
Western & Central Europe & North America	2.4	62	9	80
Middle East & North Africa	0.24	23	7	48
Global Total	40.8	1300	630	77

**Table 2:** PN-HIV intercepting hub genes, their functions, and biological functions.

Common Name	Full Name	Function	Association	References
SRC	Proto-Oncogene Tyrosine-Protein Kinase Src	Non-receptor tyrosine kinase regulating signalling pathways involved in cell–cell anchoring, survival, development, and immune response growth, survival, adhesion, and immune responses	HIV leverages Src-family kinases for enhanced viral entry, replication, and immune evasion. PN could be implicated in the modulation of SRC to inhibit these mechanisms, thereby decreasing these processes, slowing the HIV’s potential to develop infections.	[24,25]
EGFR	Epidermal Growth Factor Receptor	Receptor tyrosine kinase responsible for cell growth, multiplication, development, and cell viability	HIV glycoprotein 120 redirects EGFR pathways, thereby promoting infections and neuroinflammations. Targeting EGFR with PN promises to inhibit HIV-induced immune activation and neurotoxicity.	[26,27]
MTOR	Mechanistic Target of Rapamycin	Primary regulation of cell metabolism, development, and cellular recycling	During HIV infection, there is an activation of MTOR signalling, which assists in maintaining the viral reservoir. Therefore, the inhibition of MTOR by PN has the potential to block viral replication and improve pathogen elimination.	[28,29]
STAT3	Signal Transducer and Activator of Transcription 3	A transcription factor that regulates the immune system and signals cytokines and cell viability.	Manipulation of STAT3 by HIV promotes immune imbalance and persistent inflammation. Therefore, modulation of STA3 by PN can potentially restore immune balance and the associated HIV inflammations.	[30,31]
ESR1	Estrogen Receptor 1	A nuclear receptor that regulates the expression of genes in estrogen regulates immunity and inflammation.	Due to ESR1 immunomodulation potentials, it relatively regulates viral replication. Therefore, PN’s inhibition of the ESR1 pathway enhances immune resilience in PLHIV, especially women.	[32,33]
HSP90AA1	Heat Shock Protein 90 Alpha Family Class A Member 1	A molecular chaperone responsible for chaperoning viral and host proteins	The functioning and folding of HIV proteins depend on HSP90. Therefore, PN inhibition of HSP90 destabilizes HIV proteins and inhibits viral replication.	[34,35,36]
HIF1A	Hypoxia-Inducible Factor 1-Alpha	A transcription factor responsible for the regulation of cellular stress and hypoxic conditions	The upregulation of HIF1A by HIV promotes immune imbalance and viral latency. Therefore, PN modulation of HIF1A could result in the reduction in viral replication in a stressful environment	[37,38,39]
JUN	Jun Proto-Oncogene	A component of the AP-1 transcription factor, responsible for the regulation of cellular stress reaction, replication, and apoptosis.	The activation of JUN/AP-1 by HIV Tat and Nef proteins boosts viral transcription. Therefore, the suppression of JUN by PN could inhibit HIV replication at the transcriptional level.	[25,40,41]
AKT1	AKT Serine/Threonine Kinase 1	A central kinase in the PI3K/AKT pathway that is responsible for the regulation of cell viability, replication, and growth	Activation of AKT1 by HIV prevents cellular death in HIV-infected cells, thereby sustaining the viral reservoir. Therefore, the modulation of AKT1 by PN could improve HIV-infected cellular death and inhibit persistent viral replication	[42,43]
BCL2	B-Cell Lymphoma 2	Anti-apoptotic protein, responsible for the prevention of cellular apoptosis	The upregulation of BCL2 by HIV protects infected cells from death. Therefore, the inhibition of BCL2 by PN could facilitate the death of HIV-infected cells and lower the latent viral load	[44,45,46]

**Table 3:** PN-HIV intercepting 10 hub gene pathways selected and sorted by FDR and fold enrichment.

Enrichment FDR	Number of Genes (nGenes)	Pathway Genes	Fold Enrichment	Pathways (Biological Process)
7.8 × 10^−12^	6	79	176.6	EGFR tyrosine kinase inhibitor resistance
1.4 × 10^−13^	7	96	169.6	Endocrine resistance
1.3 × 10^−11^	6	90	155.1	PD-L1 expression and PD-1 checkpoint pathway in cancer
3.6 × 10^−11^	6	109	128	HIF-1 signalling pathway
9.8 × 10^−13^	7	138	118	Estrogen signalling pathway
6.2 × 10^−16^	9	215	97.4	Chemical carcinogenesis-receptor activation
1.0 × 10^−11^	7	203	80.2	Proteoglycans in cancer
1.1 × 10^−9^	6	194	71.9	Kaposi sarcoma-associated herpesvirus infection
1.8 × 10^−9^	6	215	64.9	Lipid and atherosclerosis
7.4 × 10^−13^	9	529	39.6	Pathways in cancer

**Table 4:** Gene Ontology Biological Process Pathways.

Enrichment FDR	nGenes	Pathway Genes	Fold Enrichment	Pathways
8.9 × 10^−8^	5	90	129.2	Regulation of miRNA transcription
8.9 × 10^−8^	5	91	127.8	Mirna transcription
9.3 × 10^−8^	5	103	112.9	Regulation of the miRNA metabolic process.
6.8 × 10^−8^	6	207	67.4	Response to reactive oxygen species
9.3 × 10^−8^	8	960	19.4	Growth
9.3 × 10^−8^	8	962	19.3	Response to hormone
1.0 × 10^−7^	8	1021	18.2	Regulation of the phosphate metabolic process.
4.1 × 10^−9^	10	1566	14.9	Response to endogenous stimulus
7.0 × 10^−9^	10	1772	13.1	Response to oxygen-containing compound
9.3 × 10^−8^	9	1621	12.9	Reg. of apoptotic proc.

**Table 5:** Gene Ontology Cellular Components Pathways.

Enrichment FDR	nGenes	Pathway Genes	Fold Enrichment	Pathways
4.4 × 10^−4^	2	9	516.8	Dendritic growth cone
7.4 × 10^−4^	2	14	332.3	Dendrite terminus
3.6 × 10^−4^	3	65	107.3	Euchromatin
4.3 × 10^−3^	2	50	93	Myelin sheath
4.3 × 10^−3^	3	286	24.4	RNA polymerase ii transcription regulator complex
8.0 × 10^−3^	3	383	18.2	Cell projection membrane
2.4 × 10^−3^	4	564	16.5	Transcription regulator complex
1.1 × 10^−2^	3	461	15.1	Endocytic vesicle
7.7 × 10^−3^	4	917	10.1	Mitochondrial envelope
4.3 × 10^−3^	5	1441	8.1	Organelle envelope

**Table 6:** Gene Ontology molecular function pathways.

Enrichment FDR	nGenes	Pathway Genes	Fold Enrichment	Pathways
2.0 × 10^−7^	3	7	996.8	Nitric-oxide synthase regulator activity
1.2 × 10^−5^	4	168	55.4	Protein phosphatase binding
2.9 × 10^−5^	4	222	41.9	Phosphatase binding
5.9 × 10^−6^	5	351	33.1	Ubiquitin protein ligase binding
6.2 × 10^−6^	5	369	31.5	Ubiquitin-like protein ligase binding
6.2 × 10^−6^	5	376	30.9	RNA polymerase II-specific DNA-binding transcription factor binding
1.0 × 10^−6^	6	527	26.5	DNA-binding transcription factor binding
2.0 × 10^−7^	7	648	25.1	Transcription factor binding
2.9 × 10^−7^	7	762	21.4	Protein kinase binding
4.3 × 10^−7^	7	843	19.3	Kinase binding

**Table 7:** Molecular docking results of PN-HIV protein complexes.

Protein	Phytochemicals	Scores (kcal/mol)	Protein	Phytochemicals	Scores (kcal/mol)
1. EGFR	Co-crystallized	−9.523	5. JUN	Co-crystallized	−10.563
	Rutin	−8.127		Catechin	−9.512
	Catechin	−7.048		Emodin	−9.033
	(-)-epicatechin	−6.579		chrysophanol	−8.902
	Quercetin	−6.560		Quercetin	−8.786
	2,3,4′,5,6-pentahydroxybenzophenone-4-C-glucoside	−6.343		quercetin-3-O-arabinoside	−8.761
2. ESR1	Co-crystallized	−11.658	6. MTOR	Co-crystallized	−4.952
	Aloin	−8.585		Rutin	−6.386
	Rutin	−8.450		quercetin-3-O-arabinoside	−6.006
	epigallocatechin gallate	−8.307		Catechin	−5.850
	Quercetin	−7.805		(-)-epicatechin	−5.741
	physcion	−7.514		quercetin-3-0-β-D-(6′-galloyl)-glucopyranoside	−5.708
3. SRC	Co-crystallized	−3.912	7. HSP90AA1	Co-crystallized	−10.030
	Quercetin	−4.520		Rutin	−10.578
	gallic acid	−4.486		7-7′-dihydroxy-3-8′-biscoumarin	−9.140
	Catechin	−4.430		2,3,4′,5,6-pentahydroxybenzophenone-4-C-glucoside	−9.103
	Emodin	−3.800		quercetin-3-0-β-D-(6′-galloyl)-glucopyranoside	−9.017
	(-)-epicatechin	−3.797		Chrysophanol	−8.695
4. AKT1	Co-crystallized	−11.385	8. STAT3	Co-crystallized	−12.045
	quercetin-3-O-arabinoside	−9.874		7-7′-dihydroxy-3-8′-biscoumarin	−6.253
	quercetin-3-0-β-D-(6′-galloyl)-glucopyranoside	−9.054		quercetin-3-0-β-D-(6′-galloyl)-glucopyranoside	−5.235
	(-)-epicatechin	−9.000		gnidimacrin	−5.049
	Rutin	−8.954		Rutin	−4.918
	epigallocatechin gallate	−8.892		Catechin	−4.904
9. HIF1A	Co-crystallized	−8.011	10 BCL2	Co-crystallized	−4.982
	Catechin	−7.153		quercetin-3-0-β-D-(6′-galloyl)-glucopyranoside	−7.021
	quercetin-3-O-arabinoside	−6.989		gnidimacrin	−6.720
	Aloin	−6.845		procyanidin B2	−5.737
	Quercetin	−6.718		(-)-epicatechin	−5.679
	Rutin	−6.642		2,3,4′,5,6-pentahydroxybenzophenone-4-C-glucoside	−5.603

**Table 8:** Detailed 2D Protein–Ligand Interaction Analysis of PN Phytochemicals with HIV Hub Proteins.

Protein	PN Ligand	Interacting Residues & Type of Bonds	Comparison with Co-Crystallized Ligand Binding Mode	Implications in HIV Pathogenesis
EGFR	Rutin	SER885, ARG889, SER921, GLY874, VAL876, ILE886, TYR891, SER924, ILE923, GLU922, ALA920, GLY873, LYS875, ILE878, LYS879, MET881. Hydrogen bonds, van der Waals, and hydrophobic interactions.	The docked PN ligand occupies the same canonical ATP-binding pocket as the co-crystallized EGFR inhibitor, forming overlapping hydrogen-bond and hydrophobic interactions with key hinge and pocket-lining residues. This spatial overlap supports a conserved binding orientation and functional relevance of the predicted pose.	Stable binding within the EGFR pocket suggests that modulation of EGFR-mediated signaling, which HIV exploits for entry, replication, and immune evasion, is possible.
HSP90AA1	Rutin	ASP91, ILE96, MET98, ASP102, LEU103, ASN106, LEU107, ILE110, ALA111, LYS58, ALA55, SER52, ASN51, GLY97, GLY117, GLY135, PHE118, TYR119, TRP162. Hydrogen bonds and hydrophobic interactions.	The PN ligand aligns within the N-terminal ATP-binding domain similarly to the co-crystallized ligand, engaging conserved residues involved in chaperone activity. Shared hydrophobic and hydrogen-bond interactions indicate preservation of the native binding architecture.	Supports disruption of HSP90AA1 chaperone activity required for HIV protein folding and replication.
JUN	Catechin	LYS92, ALA91, MET146, GLU147, LEU148, ASP150, ALA151, ASN152, LEU206, GLN155, MET149. Hydrogen bonds, hydrophobic interactions, π–π bonding.	The docked ligand binds within the transcriptionally relevant interface occupied by the co-crystallized ligand, maintaining comparable orientation and interaction patterns. This suggests effective engagement of JUN regulatory regions involved in transcriptional modulation.	Suggests inhibition of JUN-mediated transcriptional activation involved in HIV replication and immune activation.
AKT1	Quercetin-3-O-arabinoside	ASN51, ASN54, LEU210, ALA212, ILE290, ASP292, TYR272, VAL270, LYS268, SER205, THR211, TRP80, GLN79, VAL271. Hydrogen bonds and hydrophobic interactions.	The PN ligand localizes to the same kinase-associated pocket as the co-crystallized ligand, interacting with residues critical for catalytic regulation. The similarity in pose supports functional modulation rather than nonspecific surface binding.	Indicates modulation of AKT1 survival signalling used by HIV to maintain infected-cell longevity and viral reservoirs.
ESR1	Aloin	GLU352, ALA350, LEU349, THR347, MET342, ASP351, LEU346, ARG394, LEU391, MET388, LEU384, TRP383, MET421, ILE424, PHE425, LEU428. Hydrogen bonds, hydrophobic and charged interactions.	The docked ligand overlaps with the ligand-binding domain occupied by the co-crystallized estrogen receptor ligand, reproducing key polar and hydrophobic contacts that stabilize receptor–ligand complexes. This confirms biologically plausible binding.	Stable ESR1 binding suggests interference with ESR1-mediated transcriptional and immune-regulatory pathways in HIV infection.
BCL2	Quercetin-3-O-β-D-(6′-galloyl)-glucopyranoside	ALA108, ARG105, VAL92, GLU95, LEU96, ARG98, ASP99, TYR67, ASP70, PHE71, GLU73, MET74, GLN77, PHE112, GLU111, PHE109. Hydrogen bonds, hydrophobic, charged, and π–π interactions.	The PN ligand occupies the BH3-binding groove similarly to the co-crystallized ligand, engaging conserved hydrophobic and charged residues essential for anti-apoptotic function. This overlap supports competitive binding within a validated functional site.	Occupation of the BCL2 binding pocket suggests inhibition of anti-apoptotic activity, promoting apoptosis of HIV-infected cells and reducing latent viral load.

## Data Availability

The original contributions presented in this study are included in the article/Supplementary Material. Further inquiries can be directed to the corresponding authors.

## Supplementary Materials

The following supporting information can be downloaded at: https://www.mdpi.com/article/10.3390/ijms27020808/s1.
