# Supplementary material for: Molecular Investigation of Product Nkabinde in HIV Therapy: A Network Pharmacology and Molecular Docking Approach
Source: Int J Mol Sci. 2026 Jan 13;27(2):808. doi: 10.3390/ijms27020808 (PMC12841023; doi:10.3390/ijms27020808)
Supplement: Supplementary file 1 [file ijms-27-00808-s001.zip › ijms-4065293-supplementary.pdf]

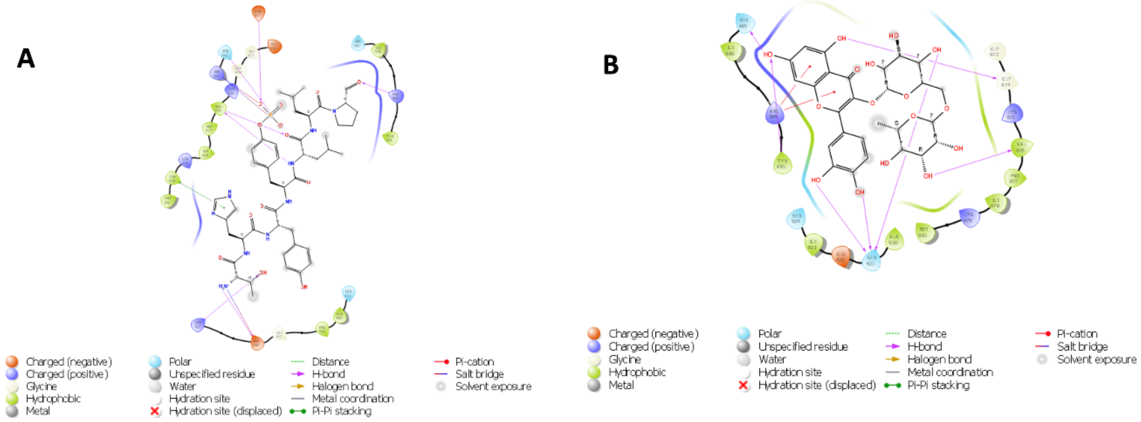

Figure S1 above represents a 2D diagram of the protein-ligand complex of the co-crystallized- EGFR (A) and the Rutin-EGFR complex (B), respectively.

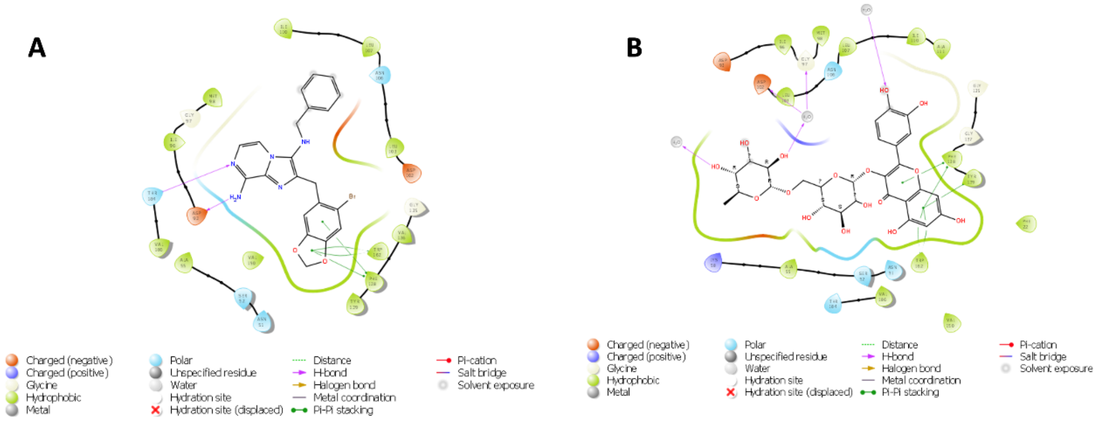

Figure S2 above represents a 2D diagram of the protein-ligand complex of the co-crystallized- HSP90AA1 (A) and the Rutin-HSP90AA1 complex (B), respectively.

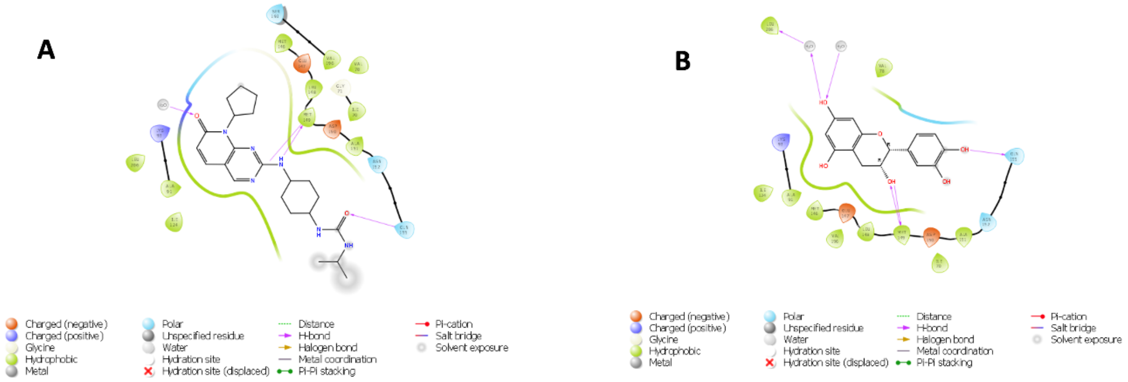

Figure S3 above represents a 2D diagram of the protein-ligand complex of the co-crystallized- JUN (A) and the Catechin-JUN complex (B), respectively.

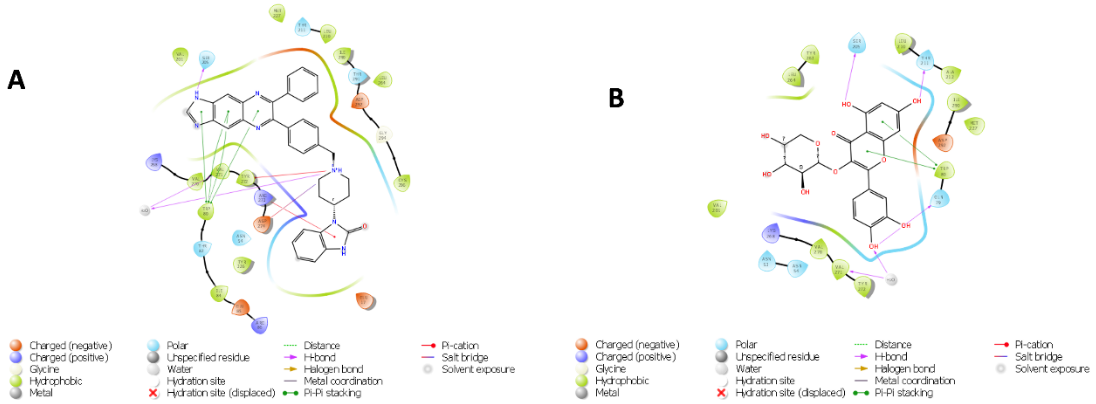

Figure S4 above represents a 2D diagram of the protein-ligand complex of the co-crystallized- AKT1 (A) and the quercetin-3-O-arabinoside-AKT1 complex (B), respectively.

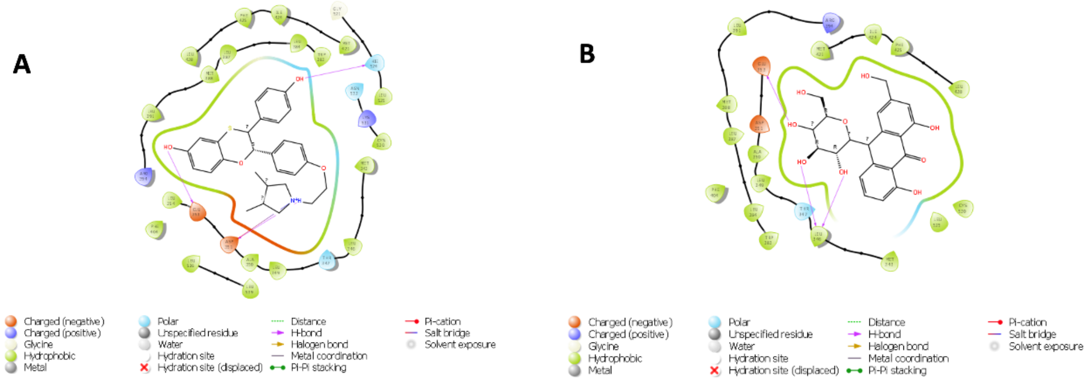

Figure S5 above represents a 2D diagram of the protein-ligand complex of the co-crystallized-ESR1 (A) and the aloin-ESR1 complex (B), respectively.

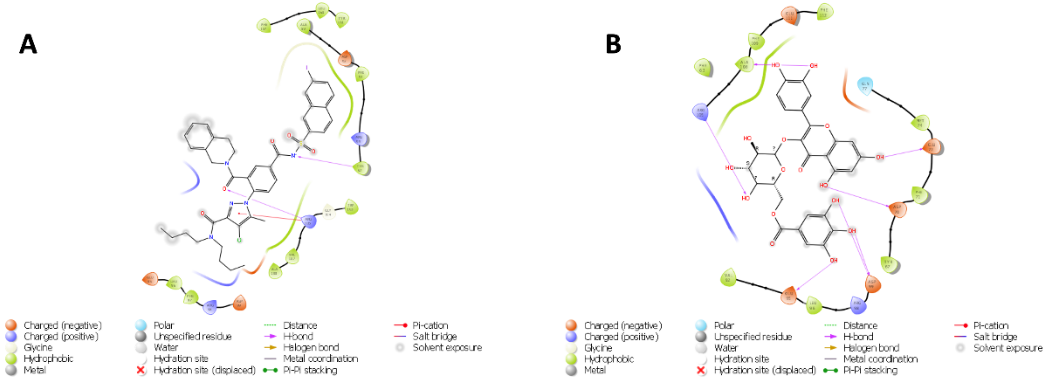

Figure S6 above represents a 2D diagram of the protein-ligand complex of the co-crystallized-BCL2(A) and the quercetin-3-0-β-D-(6’-galloyl)-glucopyranoside-BCL2 complex (B), respectively.
